# Characterizing a heterogeneous chronic patient population for redesigning person-centred bundled payment models using risk-mitigating measures

**DOI:** 10.1007/s10198-025-01762-x

**Published:** 2025-03-15

**Authors:** Sterre S. Bour, Lucas M. A. Goossens, Maureen P. M. H. Rutten-van Mölken

**Affiliations:** 1 Erasmus School of Health Policy and Management, Erasmus University Rotterdam, Rotterdam, Netherlands; 2 Institute for Medical Technology Assessment, Erasmus University Rotterdam, Rotterdam, Netherlands

**Keywords:** Bundled-payment model, Chronic disease management, Risk-adjustment, Person-centred care, Cluster analysis, Prediction models, I13

## Abstract

Since 2010, most Dutch patients with diabetes mellitus type 2 (DM2), an increased risk of cardiovascular diseases (CVR), and chronic obstructive pulmonary disease (COPD), have been treated in single-disease management programs (SDMPs) provided by primary care cooperatives (PCCs). These SDMPs are funded through bundled payments. However, given the prevalence of multimorbidity among patients, there is a growing need for care that is more person-centred. We have previously published an alternative payment model that stimulates the integration of care required for a person-centred approach and in this paper, we demonstrate an operationalization of this model. We performed cluster analysis on claims data to distinguish between different subgroups of patients, predicted cluster probabilities with data available to general practitioners, designed different care packages and investigated the expected financial risk for PCCs of different sizes. We showed that mainly the size of the PCC and the content of the care package influenced the predicted losses or profits for the PCC. Two risk-mitigating measures—risk-adjustment and cost-capping—resulted generally in predicted losses or profits closer to 0, and therefore a reduced risk for the PCC.

**Supplementary Information:**

The online version contains supplementary material available at 10.1007/s10198-025-01762-x.

## Introduction

In the Netherlands chronic care for diabetes type 2 (DM2), an increased risk of cardiovascular diseases (CVR), and chronic obstructive pulmonary disease (COPD) is mainly provided in general practitioner (GP) practices. These practices offer single-disease management programs (SDMPs) for the respective chronic diseases on a large scale since 2010 [1]. The SDMPs are reimbursed by the compulsory basic health insurance through bundled payments [2]. The SDMPs are coordinated by a primary care cooperative (PCC), an organisational entity in a specific region which mainly consists of general practitioners [3]. PCCs negotiate with health insurers about the content and price of SDMPs [4]. The SDMPs have improved quality of chronic care in terms of process indicators (e.g., if the smoking status was registered) [5, 6], but the evidence of (long-term) clinical effects of the improvement on these indicators is mixed [7–9].

SDMPs have several limitations inherent to their focus on a single disease [8, 10]. The scope and services included in the current bundled-payment model are rather limited and largely based on a one-size-fits-all approach for the provision of protocolized care. Especially for the increasing number of patients with multimorbidity (i.e., two or more chronic diseases) [11, 12], current SDMPs do not seem to be a fit [13]. Services that are not covered by the bundled payment are typically reimbursed through fee-for-service (for primary care) or diagnosis-treatment-combinations (for hospital care), paid by the health insurer. The patient pays an annual mandatory deductible of maximum €385 for care outside the GP-practice or SDMPs. There is evidence that SDMPs have led to an increase in total healthcare costs, especially for patients with multi-morbidity, probably as a result of the detection of unmet need, double declarations, and a higher referral rate of the complex patients to secondary care [13].

In response to the limitations of SDMPs, a more person-centred and integrated care (PC-IC) approach of chronic disease management in primary care could be beneficial. Several of such initiatives are currently underway in the Netherlands, among which is the OPTIMA FORMA initiative [14]. *OPTIMA FORMA* attempts to combine a holistic assessment of patient’s health and social context, with an agreement on personal and medical goals, and interventions to achieve these goals [14]. Such person-centred approaches require a matching payment model [15]. We previously sketched the possible contours of a payment model for OPTIMA FORMA. The proposed payment model consists of three parts, (1) a person-centred bundled payment—a more flexible bundled payment that allows targeting the care to the individual needs of patients with DM2, CVR and/or COPD—negotiated between the PCC and health insurer, (2) a shared savings model that compares the total (health)care expenditures of this delineated patient population with a virtual budget of expected costs, and (3) a pay-for-performance part, which determines the sharing ratio between health insurer and PCC, based on achieved results [15]. This payment model should incentivise integration and collaboration between (health)care providers that goes beyond a single disease, stimulate cost-conscious behaviour, redress incentives for risk selection [16–18], and avoid unacceptable financial risks for PCCs by appropriate risk-adjustment and cost-capping, while safeguarding quality of care [15].

With these goals in mind, we aim to operationalize the person-centred bundled-payment model for the target population of the OPTIMA FORMA initiative and investigate the risk of unacceptable losses or profits for the PCCs.

## Methods

**Fig. 1:**
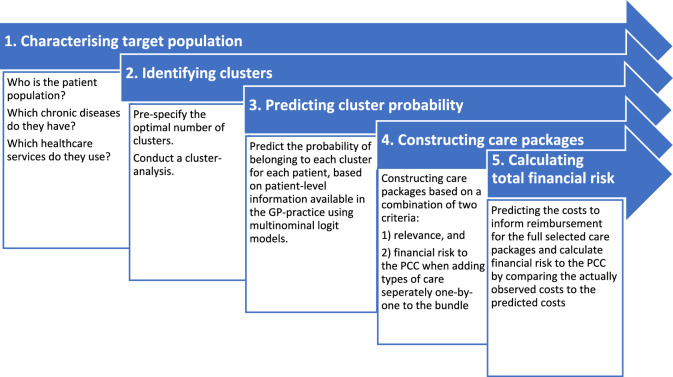
Steps taken in the method section to construct care packages and calculate the financial risk. *PCC* primary care cooperative, *GP* general practitioner

To operationalize the proposed payment model, we took the following steps. Firstly, we characterized the target population and described its heterogeneity with respect to healthcare utilization and costs. Secondly, we identified clusters of patients with relatively homogenous patterns of healthcare utilization. Thirdly, we developed a prediction model to determine to which cluster individual patients are likely to belong. Fourthly, we defined different packages of services that could be included in the person-centred bundled payment, and lastly, we assessed the risk that they would lead to unacceptable losses or profits for the PCC, which is an incentive for risk selection. Figure 1 provides an overview of the steps taken in the method.

### Step 1: Characterising target population

#### Study population

The study population consisted of 43,327 patients who participated in one or more of the chronic disease management programmes for DM2, CVR and COPD and were insured in 2017 by one of the large healthcare insurance companies in the region of the OPTIMA FORMA project [i.e., (Nijmegen, Arnhem, and Doetinchem)]. The three PCCs consisted of 1514 (small-sized group), 14,740 (medium-sized group) and 27,073 (large-sized group) patients.

#### Demographics

We obtained anonymous claims data from the healthcare insurer for the year 2017. This dataset contained the following general characteristics: age, gender, the number of days someone was insured throughout the year, inclusion in a disease management program for DM2, CVR and/or COPD, if someone died in 2017, if someone had a supplementary health insurance, and if the GP practice was based in a deprived area.

#### Chronic diseases

Each persons’ chronic diseases were identified based on the Anatomical Therapeutic Chemical (ATC) codes of dispensed medication and on participation in SDMPs. The ATC-3-codes of the medication were matched to a list of 22 chronic diseases published by Huber et al. [19]. We used the daily defined doses (DDDs) to label the medication intake as “chronic” when the number of prescribed days was higher or equal to 90 days per year [20].

#### Healthcare utilization

Based on claims data, we analysed the presence or absence of healthcare utilization for each of the following sectors or services: primary care in the GP practice, paramedical care, stop smoking support modules, basic mental healthcare, specialised mental healthcare, primary care inpatient stays (short hospital stays for patients who don’t have an indication for a hospital stay but cannot stay at home after, for instance, a surgery), pharmaceuticals, rehabilitation care, medical aids, medical specialist care, transport to the hospital, district nursing, and other sectors.^1^ For each of the sectors, we studied the descriptive statistics of the healthcare expenditure and the association between expenditure and number of chronic diseases.

### Step 2: identifying clusters

In the second step, we performed a non-hierarchical k-means cluster analysis to distinguish subgroups of patients with relatively homogenous healthcare use [21]. All patients were allocated to one of the clusters, based on 52 dichotomous variables describing the healthcare use (See Appendix A). To avoid noise from the cluster analysis [22], we decided to only incorporate health services that were used by more than 2% of the population (*except for stop smoking modules*), and to combine several healthcare services that were closely related (e.g., consultation and home visit of a practice nurse for mental health). The similarity of the healthcare utilization within the clusters was measured by the Jaccard coefficient [21].

We used the following stepwise approach to determine the pre-specified optimal number of clusters. Firstly, we applied the elbow method which displays the association between the number of clusters and the within-cluster sum of squares (WSS) [23]. The lower the WSS, the greater the similarity among patients within a cluster. The optimal number of clusters is the number at which the WSS does not decrease much anymore. We determined this by studying the proportional reduction of error (PRE). The PRE provides insight in the reduction of the WSS compared to the previous WSS [24]. Secondly, we investigated the stability of the clusters, when using five different seed values [25]. Stability is achieved when seed values of the stochastic clustering process have limited effect on the assignment of patients to the clusters. The stability was estimated for the preferred number of clusters according to the WSS and PRE. Finally, we discussed the interpretability of the content of the determined optimal number of clusters, i.e., whether the clusters were sufficiently distinctive and practically usable.

### Step 3: predicting cluster-probabilities

To inform the care package and the payment scheme, PCCs need to be able to predict upfront to which cluster a patient belongs. They will need to do so with less variables than available in the claims data and these variables have to be routinely available in primary care. Therefore, the third step was to develop multinominal logit (MNL) models according to the TRIPOD guideline (Transparent Reporting of a multivariable prediction model for individual Prognosis Or Diagnosis) to predict cluster-probabilities using several sets of variables [26]. For each patient, the estimated MNL-model was used to predict the probability of membership of each cluster.

The MNL-model is specified as a probability model, with cluster 1 as the reference category [27]:

$$\mathit{Pr}\left({y}_{i}=1|{x}_{i}\right)=\frac{1}{1+ {\sum }_{j=2}^{J}\text{exp}({x}_{i}{\beta }_{j})}\quad \text{Since},\text{ exp}\left({x}_{i}{\beta }_{1}\right)=\text{exp}\left({x}_{i}0\right)=1$$

$$\mathit{Pr}\left({y}_{i}=m|{x}_{i}\right)=\frac{\text{exp}\left({x}_{i}{\beta }_{m}\right)}{1+ {\sum }_{j=2}^{J}\text{exp}({x}_{i}{\beta }_{j})}\quad  for\,\, m>1$$

In these equations, ${y}_{i}$ is an individual’s cluster, *m is the number of a cluster,* ${x}_{i}$ are the independent variables, ${\beta }_{m}$ is a vector of coefficients for clusters *m* and ${\beta }_{j}$ is a vector of coefficients for all clusters [27].

Since we assumed that the information that would be available at different GP practices could vary, we developed seven different MNL models with an increasing number of independent variables:

- Model 1: Simple (7 variables): Gender, age, deprived area, additional insurance, SDMPs (DM2, CVR, COPD).
- Model 2: District nursing (8 variables): Gender, age, deprived area, additional insurance, SDMPs (DM2, CVR, COPD), and usage of district nursing.
- Model 3: Chronic medication (19 variables): Gender, age, deprived area, additional insurance, SDMPs, other chronic diseases (a list of 12 chronic disease^2^ variables that were present in ≥ 3% of the study population).
- Model 4: Specialist care (20 variables): Gender, age, deprived area, additional insurance, SDMPs, and utilization of specialist care categories (a list of 13 different medical specialists^3^) that are used by ≥ 3% of the study population).
- Model 5: District nursing + chronic medication (20 variables): Gender, age, deprived area, additional insurance, SDMPs, district nursing, and 12 chronic diseases.
- Model 6: District nursing + specialist care (21 variables): Gender, age, deprived area, additional insurance, SDMPs, district nursing, and usage of 13 medical specialists.
- Model 7: District nursing + chronic medication + specialist care (33 variables): Gender, age, deprived area, additional insurance, SDMPs, district nursing, 12 chronic diseases, and usage of 13 medical specialists.

We developed the prediction models with all available data of the three PCCs (n = 43,327) and validated the models by estimating the performance for each PCC separately. The performance of the MNL-models was assessed with calibration, discrimination, and classification according to the TRIPOD guideline [26].

Calibration is the comparison between the observed proportion and the predicted proportion (the calibration-in-the-large measure), expressed with calibration plots and recalibrated if necessary [28].

Firstly, calibration-in-the-large was measured for each PCC. A lower calibration-in-the-large estimate indicates more similarity between the observed and predicted probabilities. We checked whether it was necessary to recalibrate the originally developed model in order to improve the fit for the local settings [29].

Secondly, discrimination was measured as the degree to which the model can distinguish between patients who belong to a certain cluster and patients who do not belong to that cluster. Discrimination was expressed in the polytomous discrimination index (PDI), which is an extension of the c (concordance)-index, proposed by Van Calster et al. [30]. The PDI compares different sets of six patients (from each cluster) and compares their predicted probabilities. The PDI can take a probabilistic value between 0.167 (1 divided by the number of categories) and 1 (perfect discrimination). We estimated the overall PDI for the developed model and for each PCC separately.

Lastly, we assessed the classification of the different MNL-models using sensitivity and specificity, based on the highest predicted probability, defined as:

- Sensitivity = the proportion of people from a certain cluster who were predicted to be in that cluster based on the highest predicted probability.
- Specificity = the proportion of people who were not from a certain cluster who were predicted not to belong to that cluster.

We used STATA/MP 18.0 to do the analyses and downloaded two additional packages to estimate the PDI [31] and the calibration plots [32].

### Step 4: constructing care packages

In the fourth step, we constructed different care packages (i.e., bundles of services). These packages were constructed based on two criteria:

1. The “relevance” for the provision of person-centred care by removing barriers for cooperation between providers. Relevance was based on (1) the patterns of healthcare use in the clusters of the cluster analysis, (2) if the care was mentioned in the clinical guidelines for the respective chronic diseases [33–35], and (3) on the percentage of patients that used a certain type of care. A certain type of care was considered to be highly relevant when it was mentioned in one of the clinical guidelines and if more than 10% of the total target population used that care. If one of these two conditions was met and when it matched the patterns of care found in one of the clusters of the cluster analysis, we qualified the care as being of high relevance. If it did not match the patterns of care found in the clusters, we qualified the care as being of medium relevance. If none of the conditions were met, the type of care was classified as being of low relevance (see Appendix F for the assessment). For the constructed care packages, we only included highly relevant care.
2. “The “financial risk” to the PCC of adding types of care separately one-by-one to the bundled-payment model. Risk was based on the estimated losses (or profits) for PCCs when comparing the predicted costs with the actual observed costs of a constructed bundle.

The predicted costs were based on a linear regression model. The dependent variable was the per-patient annual cost of a care within a bundle. These costs would vary across patients, because they would not all use the same amount of care that is available within the bundle. The independent variables were the predicted probabilities of patients of belonging to each of the clusters. The regression results were used to calculate predicted costs per patient, based on their cluster membership probabilities. These patient-level predicted costs for a constructed bundle were averaged over all patients of a PCC to calculate the mean costs per patient for a PCC.

Risks for PCC result from the unpredictability of the per-patient costs, which could incentivise risk selection. We defined a risk as low if the annual loss or profit per patient was lower than €10, medium if the loss was between €10-€30, and high if the loss was more than €30 per patient. Appendix G shows all the estimated profits and losses per type of care and per PCC. To construct the care packages, we defined a certain type of care as being of low risk when the annual loss or profit was lower than €10 for at least two of the three PCCs.

### Step 5: calculating the financial risk

To calculate the total financial risk of each defined care package, we subtracted the observed costs of the defined care package from the predicted costs for the defined care package. In practice, the predicted costs for a defined care package could be used for negotiations between the health insurer and the PCC about the future reimbursement of a particular care package. We compared the financial risk for the PCC in different scenarios: with and without case-mix adjustment combined with or without cost-capping. The predicted costs for a care package were calculated in the same way as the “financial risk” to the PCC when adding a single type of care to the bundled-payment model. In this case, the dependent variable was the cost of care of a constructed care package at the level of individual patients. The independent variables were the predicted probabilities of patients of belonging to each of the clusters. For the case-mix risk adjustment, the probability of belonging to each of the clusters was based on the optimal MNL-model. For the analysis without case-mix adjustment, we simply calculated the predicted costs assuming that the proportions of patients per cluster (as determined in the cluster analysis), would be similar for each PCC.

We also calculated the total financial risk for the PCC while capping the total costs of a defined care package at the 99th percentile and at the 95th percentile. This means the top 1% or 5% expenditures are not reimbursed through the bundled payment but through the conventional payment system.

## Results

### Characterising target population

The claims dataset consisted of 43,327 patients, whose characteristics are shown in Table 1. Most people are included in the SDMP for CVR (57%) and the three most common chronic diseases were CVR (including hypertension), hyperlipidaemia, and acid related disorders.

**Table 1:** Characteristics of study population

Category	Number of patients n = 43,327 (%)	Category	Number of patients n = 43,327 (%)
Female	23,749 (55%)	Chronic disease based on chronic medication usage	
Average age (interquartile)	67.6 (16)	Acid related disorders	16,293 (38%)
Age groups		Bone diseases (osteoporosis)	1308 (3%)
18–30	86 (0.2%)	Cancer	3 (0.01%)
31–40	470 (1%)	Cardiovascular diseases (incl. hypertension)	32,936 (76%)
41–50	2,844 (7%)	Dementia	105 (0.2%)
51–60	8,293 (19%)	Diabetes mellitus	11,478 (26%)
61–70	13,596 (31%)	Epilepsy	1271 (3%)
71–80	12,003 (28%)	Glaucoma	1495 (3%)
80+	6035 (14%)	Gout, hyperuricemia	1381 (3%)
Supplemental insurance	39,029 (90%)	Human immunodeficiency virus (HIV)	47 (0.1%)
Did not pass away in 2017	42,370 (98%)	Hyperlipidaemia	24,012 (55%)
Living in a deprived area	1944 (4%)	Intestinal inflammatory diseases	301 (1%)
SDMPs		Iron deficiency anaemia	359 (1%)
DM2	14,764 (34%)	Migraines	173 (0.4%)
CVR	24,507 (57%)	Pain	2169 (5%)
COPD	1365 (3%)	Parkinson’s disease	324 (1%)
Multiple SDMPs	2691 (6%)	Psychological disorders (sleep disorder, depression)	4964 (11%)
Chronic diseases based on chronic medication and SDMP		Psychoses	770 (2%)
1	8585 (20%)	Respiratory illness (asthma/COPD)	6624 (15%)
2	11,441 (26%)	Rheumatologic conditions	2425 (6%)
3	10,481 (24%)	Thyroid disorders	3028 (7%)
4	7109 (16%)	Tuberculosis	8 (0.02%)
5+	5711 (13%)		

SDMPs = single disease management programs, DM2 = diabetes mellitus type 2, CVR = cardiovascular risk, COPD = chronic obstructive pulmonary disease

Table 2 shows that the highest mean costs were for medical specialist care, district nursing and medication. Distributions were very skewed for medication and specialist healthcare costs, with a small number of patients having extremely high costs. The mean costs for the currently used bundled payments are €238, based on the weighted average costs of the currently used bundled payments (mean of the bundle for DM2 (mean = €359), CVR (mean = €142) and COPD (mean = €224)) plus the overhead costs for the PCC (€4).

**Table 2:** Average healthcare costs per sector for users of the category of healthcare

Category of healthcare	Mean costs	Users, n (%)	Mean, per user	Median, per user	First quartile	Third quartile
Medical specialist care (DRGs)^a^	€2282	26,657 (62%)	€3574	€1160	€445	€3353
District nursing^b^	€866	5783 (13%)	€6488	€3228	€781	€9308
Pharmaceuticals	€743	42,533 (98%)	€757	€395	€163	€952
Medical specialist care (other)	€530	39,383 (91%)	€583	€95	€44	€199
Medical aids (from basic health insurance package)	€247	16,226 (37%)	€660	€272	€115	€716
General practitioner (S2^c^)	€238	43,327 (100%)	€238	€164	€140	€360
Specialist mental health care	€223	1351 (3%)	€7136	€2,516	€1121	€5236
General practitioner (S1^c^)	€186	43,327 (100%)	€186	€153	€117	€214
Paramedical (from additional health insurance package)	€143	15,417 (40%)	€403	€306	€162	€554
Rehabilitation care	€112	349 (1%)	€13,850	€11,255	€7444	€19,173
Transport to hospital	€86	3888 (9%)	€963	€733	€702	€1024
Paramedical (from basic health insurance package)	€86	4695 (11%)	€798	€382	€150	€1105
Primary care inpatient stays	€63	319 (1%)	€8506	€6795	€3043	€11,466
General practitioner (other)	€35	18,197 (42%)	€84	€44	€10	€102
Other sectors	€30	3716 (9%)	€354	€211	€105	€353
Medical aids (from additional health insurance package)	€19	6831 (16%)	€122	€100	€100	€150
General practitioner (S3^c^)	€14	42,619 (98%)	€14	€8	€5	€10
Basic mental health care	€10	438 (1%)	€1006	€1146	€757	€1238
Stop smoking support modules	€2	547 (1%)	€187	€167	€88	€253
Total	€5917	43,327 (100%)	€5917	€2129	€961	€5403

*DRGs* diagnosis related groups

^a^Without obstetrics and paediatrics

^b^Without performance payment for district nursing

^c^S1 = the capitation payment for each contracted patient plus the services that are reimbursed fee for service. S2 = the bundled payments for the chronic disease (DM2, CVR, COPD), and S3 = payments for innovations and quality of care

**Fig. 2:**
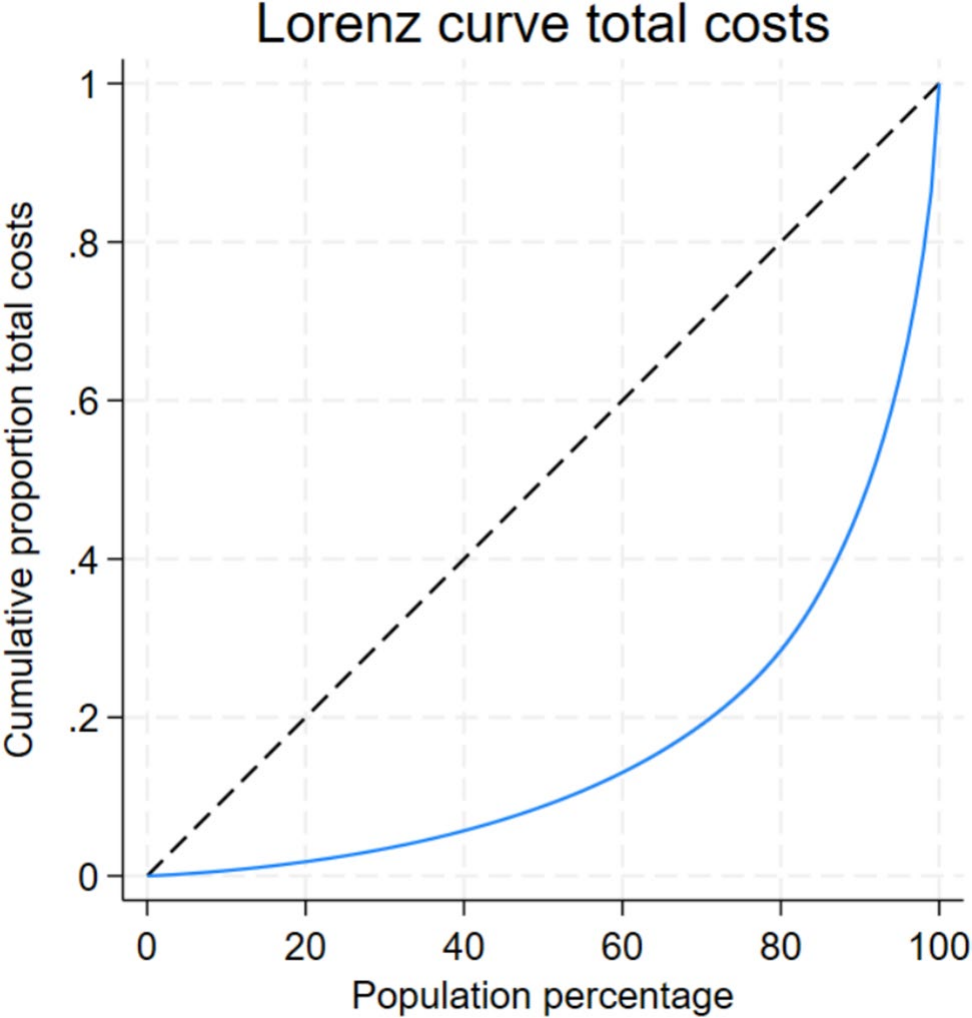
Cumulative distribution from the total healthcare expenditures

There is an increase in the mean costs, with a greater increase for each additional chronic disease in one patient (see Appendix B). Figure 2 shows the Lorenz curve of the total healthcare costs, which are unequally distributed over the study population. The top 20% of the population accounts for more than 75% of the total healthcare expenditures.

### Identifying clusters

**Fig. 3:**
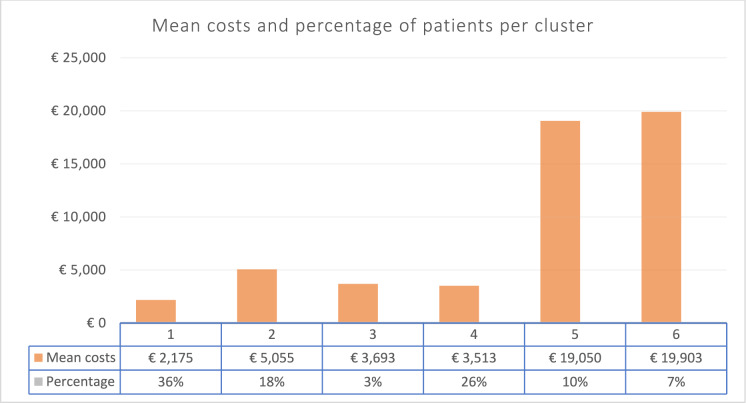
Mean costs per cluster and percentage of patients per cluster

Based on the WSS and PRE criteria, the five, six and eight-cluster solutions seemed to be the most appropriate (see figure A in Appendix C). As stability was better for the six-cluster solution (see tables C, D, and E in Appendix C) and this solution led to well-interpretable clusters, we continued the analysis with six clusters. In Fig. 3 an overview is given of the mean costs per cluster and the percentage of patients that belongs to a cluster.

A summary of the distinctive features of the clusters is given in Box 1. Detailed tables with the characteristics and healthcare utilization of the patients in the different clusters can be found in in Appendix D.

Box 1: Healthcare utilization and costs for different patient clusters Cluster 1: Relatively healthy CVR-patients with either one or two chronic diseases (n = 15,667 (36%)).

Cluster 1 patients were found to use relatively little healthcare. In this cluster, 83% of the patients used chronic medication for CVR and/or hyperlipidemia. Most patients only used primary care, and more than 50% of the people did not see any medical specialist in 2017. The mean costs for district nursing and medical aids were relatively low. The total mean healthcare costs for cluster 1 were €2175 (SD = 6,329).

Cluster 2: CVR-patients with three or more chronic diseases (n = 7871 (18%)).

Most patients in cluster 2 used medication for acid related disorders (96%), CVR (87%), hyperlipidemia (63%), respiratory illness (20%), and/or mental health issues (16%). Besides, regular consultations of the GP, a relatively high number of patients had home visits by the GP and used the evening-night-and weekend-services. A relatively high number of patients received physiotherapy both from the basic and additional insurance package. A relatively high percentage of patients utilized footcare. The patients who did use medical specialist care mainly visited the cardiologist (21%). The total mean costs for patients in cluster 2 were €5055 (SD = 7770).

Cluster 3: Relatively young patients with COPD, who mainly use chronic medication for respiratory illness (n = 1296 (3%)).

Most patients in cluster 3 had one chronic disease. Besides medication for respiratory illness (74%), people used medication for CVR (19%) and mental health issues (16%). Most people used care from the GP-practice and relatively more people tried to stop smoking than in the other clusters. The patients who did utilize medical specialist care mainly consulted the pulmonologist (11%). The mean total costs for cluster 3 were €3,693 (SD = 8639).

Cluster 4: DM2-patients with three or more chronic diseases (n = 11,397 (26%)).

In cluster 4, relatively more people were living in a deprived area (6%). Most patients used chronic medication for diabetes mellitus (74%), CVR (74%), hyperlipidemia (73%), and/or acid related disorders (35%). High costs were made in the GP-practice, especially for chronic disease management programs. In cluster 4 for almost all patients (95%), diagnostics were performed. Cluster 4 had a relatively small proportion of people using secondary care, but the specialists that were seen most were ophthalmologists (20%) and cardiologists (15%). Medical aids for diabetes were relatively often (17%) used. The mean total costs for cluster 4 were €3513 (SD = 5423).

Cluster 5: Older DM2 patients with three or more chronic diseases (n = 4185 (10%)).

Cluster 5 had a relatively high mortality rate of 10% in 2017. Most patients used chronic medication for CVR (86%), acid related disorders (73%), DM2 (70%), hyperlipidemia (65%), respiratory illness (28%), mental health issues (21%), and/or chronic pain (16%). Patients were regularly seen in the GP-practice, were visited at home, or needed intensive GP-care during the day and/or used evening-night-and weekend-services. Relatively many patients used paramedical care: physiotherapy, occupational therapy, footcare and nutritional advice. Almost all patients used medical specialist care, mostly from the cardiologist (44%), the internist (43%), and a surgeon (33%). A lot of medical aids from the basic insurance package were used by the patients like incontinence materials, urination aids, aids for diabetes, and compression stockings. Elderly care, including district nursing, rehabilitation, and a short-term hospital bed in primary care, made a substantial contribution to the high mean total costs. The mean total costs for cluster 5 were €19,050 (SD = 19,240).

Cluster 6: Older female CVR-patients, who had the highest costs for secondary care (n = 2911 (7%)).

Cluster 6 had a relatively high mortality rate of 12% in 2017. Most people used medication for two or three chronic diseases. Mainly, medication was used for CVR (81%), acid related disorders (52%), hyperlipidemia (38%), respiratory illness (32%), mental health issues (19%), and/or chronic pain (15%). Patients were regularly seen in the GP-practice, were visited at home, or needed intensive GP-care during the day, and evening-night-and weekend-services. Besides, relatively more patients used the practice nurse with a specialization in mental health and specialized mental care in comparison to the other groups. Many people used specialist care, mostly the cardiologist (36%), surgeon (36%), internist (35%), pulmonologist (29%), and neurologist (28%). A high percentage of people used physiotherapy from the basic and additional insurance package and occupational therapy, footcare, and nutritional advice. Elderly care, including district nursing (68%), rehabilitation, and a short-term hospital bed in primary care, made a substantial contribution to the high mean total costs. These aids were used the most: incontinence materials, urination aids, furnishing elements for assisted living at home, and compression stockings. The total mean costs for cluster 6 were €19,903 (SD = 19,016).

### Predicting cluster membership

Our prediction model 5 appeared to be the optimal model. It scored well on the prediction performance measures (calibration-in-the-large, polytomous discrimination index, see appendix E). The model includes variables on basic characteristics of the patients (gender, age, if the patient lives in a deprived area, if the patient has an additional insurance package, and the SDMPs a patient is participating in) plus district nursing and chronic medication. One other model (model 7) performed slightly better, but it requires much more data. Since the prediction model shows similar results in each local setting, no recalibration of the model was considered necessary.

We also assessed the risk of risk selection by evaluating the predictions on the individual level. The classification (sensitivity, specificity, see appendix E) for model 5 was good for clusters 1 to 4 but the model could not perfectly predict members from clusters 5 and 6, the most expensive clusters in which people need relatively much care.

### Constructing care packages

In Appendix F we determined the relevance of including specific services in the care package that would be covered by the bundled payment. In Appendix G we estimated the loss or profit for the PCCs by comparing the predicted costs with the mean actual costs, when adding single services to the bundle. We did so for the small (PCC 1), medium (PCC 2) and large (PCC 3) PCC, with 1,514, 14,740 and 27,073 patients, respectively. Based on the findings in appendices F and G we defined three different bundles of care in which only highly relevant care was included:

1. The low-risk and high-relevance care package consists of *general practice care, foot therapy, ergotherapy, dietician, practice therapy, stop smoking modules, basic mental healthcare, aids, ophthalmology, cardiology, neurology, and cardiopulmonary.*
2. The OPTIMA FORMA care package in which healthcare is included that is provided in the OPTIMA FORMA project. This care package consists of *general practice care, physiotherapy, foot therapy, ergotherapy, dietician, practice therapy, stop smoking modules, basic mental healthcare, medication, and district nursing*.
3. The high-relevance care package in which all care is included with a high relevance score, irrespective of the ‘risk’. This package consists of *general practice care, physiotherapy, foot therapy, ergotherapy, dietician, practice therapy, stop smoking modules, basic mental care, medication, medical aids, district nursing, ophthalmology, surgery, internal medicine, cardiology, pulmonologist, rehabilitation, neurology, cardiopulmonary and diagnostics.*

Table 4 shows the estimated losses or profits per PCC, which we would like to be close to 0 to avoid the PCCs to make a loss or a profit based on ‘wrongly’ predicted costs. We estimated these using model 5, and compared the results to the situation in which no case-mix adjustment was applied.

**Table 4:** Estimated profits or losses per patient by size of primary care cooperative for the three different care packages

Care packages	Mean observed costs per patient	Primary care cooperative	Predicted loss or profit per patient per primary care cooperative	
			No risk adjustment: mean (SD^a^)	Risk-adjusted model 5: mean (SD^a^)
No cost capping				
1. Low-risk and high-relevance care package	€1286	Small Medium Large	€− 126 (3311) €25 (3100) €− 6 (2880)	€− 180 (3199) €− 2 (2980) €11 (2774)
2. OPTIMA FORMA care package	€2216	Small Medium Large	€165 (4764) €7 (4726) €− 13 (4902)	€− 92 (4039) €1 (3691) €5 (4139)
3. High-relevance care package	€4078	Small Medium Large	€227 (7456) €135 (7669) €− 86 (7789)	€− 153 (6373) €93 (6287) €− 42 (6647)
Cost capping at 1%				
1. Low-risk and high-relevance care package	€1206	Small Medium Large	€− 97 (2317) €26 (2037) €− 9 (2082)	€− 143 (2187) €1 (1894) €7 (1959)
2. OPTIMA FORMA care package	€2094	Small Medium Large	€198 (3356) €5 (3797) €− 14 (3583)	€− 35 (2586) €− 5 (2718) €5 (2685)
3. High-relevance care package	€3907	Small Medium Large	€208 (6373) €130 (6301) €− 82 (6383)	€− 148 (5268) €88 (4800) €− 40 (5154)
Cost capping at 5%				
1. Low-risk and high-relevance care package	€1024	Small Medium Large	€− 40 (1245) €14 (1220) €− 5 (1201)	€− 71 (1128) €− 6 (1076) €7 (1082)
2. OPTIMA FORMA care package	€1734	Small Medium Large	€136 (1916) €40 (2158) €− 29 (2097)	€1 (1350) €22 (1336) €− 12 (1397)
3. High-relevance care package	€3435	Small Medium Large	€226 (4362) €113 (4517) €− 74 (4533)	€− 32 (3469) €68 (32,493) €− 35 (3481)

^a^Sd standard deviation which provides insight in the variance of the loss or profit at the patient level within the primary care cooperative

Table 4 further shows that for the small PCC, each of the three care packages results in a substantial expected loss or profit per patient, regardless of the use of case-mix adjustment and/or cost capping. For medium and large-sized PCCs, a similar pattern was observed, the loss or profit is small for the low-risk and high-relevance, and the OPTIMA FORMA care packages but not for the high-relevance care package. Risk-adjustment with model 5 seems to lower the estimated losses or profits but does not fully eliminate it. Cost-capping reduces the expected losses and profits, especially of the high-relevance care package. As expected, the loss/profit-reduction is greater for the 5% than for the 1% cost capping. However, overall cost-capping had a minor impact on the predicted losses or profits in comparison to no cost-capping probably due to the relatively small differences in observed costs per patient. Besides, risk adjustment did bring the expected loss or profit in general closer to 0. However, risk adjustment could change a profit in a loss or the other way around, which suggests the risk-adjustment model overcorrects the costs for certain patients.

## Discussion

The enrollment of patients into the SDMP for DM2, CVR and COPD that are offered by PCCs in the Netherlands was very successful [13], but the chronic care that is provided is narrow in scope, highly standardized, and not well-matched to the individual needs of patients, especially those with multi-morbidity. A more person-centred and integrated care approach seems an appropriate response, but this requires an adjustment of the current bundled-payment model. Such an alternative payment model should stimulate better collaboration between healthcare providers in the region, when needed, while at the same time incentivizing efficiency, cost-conscious behaviour [15], and safeguarding the financial risk for PCCs [13]. For many PCCs, the population that currently participates in the SDMPs is a good starting point for broadening the scope of services provided. In this paper, we demonstrated a method that could be used to develop a more person-centred bundled-payment model. Our work showed that patient-level cluster prediction can achieve a good performance in the Dutch setting of patients that are currently enrolled in SDMPs, despite the highly skewed distribution of healthcare expenditures. Assessing the use of these individual-level cluster prediction models to predict healthcare expenditure at the PCC-level is important to inform the PCC of the risks associated with budget allocation based on this alternative payment model and the need to apply financial risk-mitigation measures at the PCC-level. We report how the amount of risk for the PCC depends on the type of services included in the care package, with the addition of secondary care services (in particular: surgery, internal medicine, rehabilitation, and diagnostics) increasing the financial risk. The risk is larger for the smaller PCC, but the risk can be mitigated to some extent by risk adjustment and cost-capping at the level of the PCC.

Since a cluster analysis is inherently sensitive to the selection of variables, the predefined number of clusters, the similarity/dissimilarity coefficient, and the random starting values of a non-hierarchical cluster analysis, the study population could have been partitioned differently [21, 36, 37]. However, we made our choices with caution and the six clusters from our analysis are distinctive and meaningful. The partitioning of other PCCs could result in different clusters, depending on their patient population. The strength of our paper is the demonstration of a method how to do it. When a PCC has all information on the healthcare use of their patients, the PCC can estimate clusters themselves, create a prediction model and estimate the costs for a certain care package. However, Dutch PCCs generally have limited data (data such as used in our prediction models), in which case the PCC can use our clustering and estimate the costs depending on the proportioning of patients.

In this specific case, we found that a prediction model based on patient characteristics, medication use, and use of district nursing (MNL-model 5) was optimal to predict patient cluster probabilities upfront. To expand the bundled-payment model, the covered size of the PCC (number of registered patients) needs to be medium-sized or large to avoid high losses or profits. The low-risk-high-relevance care package and the OPTIMA FORMA care package seem feasible to implement for the medium- and large-sized PCCs. However, the costs of the high-relevance care package appear to be unpredictable, regardless of the size of the PCC. The risk mitigating measures, i.e., risk adjustment based on predicted cluster probabilities and cost capping at 1% or 5%, reduce the profits or losses, bringing the estimates in general closer to 0 for all PCCs and care packages but the impact of these measures is limited.

For the alterative payment model to work, it is important that any profits or losses depend on how the PCC performs in terms of quality and efficiency, and not on badly predicted costs, risk selection or under-provision of care. We showed that incentives for risk selection cannot be fully eliminated, since the classification of each prediction model does not result in an accuracy of 100% for all clusters (see Appendix E). PCCs especially have to assess the patients predicted in clusters 1 (mostly relatively healthy CVR patient) or 2 (mostly CVR patients with multiple chronic conditions) with caution because some of them actually belong to cluster 6 (mostly older DM2 patients with high medication cost). Furthermore, some patients predicted to belong to cluster 4 (mostly relatively young COPD patients), possibly belong to cluster 5 (mostly DM2 patients with multiple chronic diseases). The patients of clusters 5 and 6 generally need more care, so not identifying those patients could result in undertreatment or underpayment for these patients. This could result in potential losses for the PCC. Hypothetically, the PCC could deliberately avoid the enrollment of patients who are difficult to predict in their patient population. The risk mitigation measures in our study had some effect but did not seem to have a large impact on the estimated profits and losses. Possibly, innovative risk mitigating measures like residual-based risk sharing could be explored to reduce excessive over-or under-payments and risk selection [17]. In residual-based risk sharing, PCCs would receive additional money for individuals who were heavily underpaid and would redraw money for those individuals who were heavily overpaid by the risk-adjusted payment model (residual-based repayments) [38, 39].

### Limitations

Firstly, data on the use of social support services were not available in the dataset. That would have been very relevant since the PC-IC programme of OPTIMA FORMA stimulates the primary care physicians to also investigate the social support need of a patient [14], which could possibly require referral to and coordination with social support services. However, integration of health and social services in the Netherlands is challenging due to the fragmented finance systems. Health care is regulated by the Health Insurance Act and financed by health insurers. Social support is regulated by the Social Support Act and largely financed by the municipalities. Our recommendation is to initially expand the SDMPs with services financed through the Health Insurance Act, and possibly expand the packages with social services in a later stage.

Secondly, we used chronic medication use as a proxy for the identification of chronic diseases. Although this proxy is commonly used, it could have led to misclassifications due to medication prescribed for a different reason (i.e., medication for rheumatoid arthritis is also used as pain medication), patients with a chronic disease who were not treated with pharmaceuticals, or patients who failed to fill their medical prescriptions at the pharmacy. However, by requiring that the medication should be used for more than 3 months, we disregarded medication for acute disease.

Thirdly, the constructed care packages were designed based on data about healthcare use and costs of 2017, including the use of SDMPs. If the alternative payment model were to be implemented, SDMPs would no longer exist, and other incentives for efficiency would be in place. In this case, cluster predictions could be based on diagnosis of chronic diseases instead of SDMP-use, and the constructed care packages might have to be adapted to the changed medical practice. Therefore, the alternative payment model requires regular updating.

Lastly, we only had data of three PCCs, which leads to concerns about the external validity of the results. The PCCs in this analysis are all from one region. Preferably, we would have had data from all PCCs in the Netherlands to apply this method. Besides, it would have also been better if we would have had data from multiple years to check if the result per PCC changes over time. However, we had data of a small, medium and large PCC and were still able to provide good insight in the (un)predictability of the costs for different care packages, which provides valuable insight in the consequences of implementing the alternative payment model for these PCCs. Besides, we provided an approach of five steps which could be applied to any local setting. The cluster analysis of this patient population could be repeated with locally relevant predictors and the probability of belonging to each cluster should be predicted in the local setting.

### Implications

The most important implication of this study is that the coordinators of the PCCs should make sure that if they broaden the scope of the bundled-payment models for people with chronic diseases, the number of patients covered through the bundled payment is large enough. We showed that the predictability of costs is highly dependent on the size of the PCC. Besides, it is important that the PCC and the health insurer decide on which services to include in the care package and finance through the bundled-payment model.

The implication to broaden the bundled payment is mainly challenging due to the highly fragmentated health care system of the Netherlands in terms of finance and organization between primary care, secondary care, social services, and long-term care [40, 41]. It would require a fundamental change in the system to integrate these budgets. In the absence of this integration, the PCCs need to demonstrate strong leadership to overcome the fragmentation by coordinating and aligning the various (health)care providers and social support organizations [42]. Besides, the PCC should enable data sharing and facilitate interoperability between health information systems of different professions that enables effective coordination of care and collaboration between different (healthcare) organizations [42]. A promising development is the 2022/2023 Cross-sectoral Care Agreement (Integraal ZorgAkkoord, IZA) that was reached between all relevant stakeholders in the Netherlands, including amongst others primary, secondary, and tertiary health and social care providers, insurers, municipalities, public health and patient organizations [43]. It is a typical Dutch way of addressing the persistent challenges around appropriate care (right care in the right place), rising health expenditure, shortage of staff and climate change. Operationally, by bringing all stakeholders on board, the IZA will lead to many regional plans to strengthen the collaboration between stakeholders in the region with financial support from the Ministry of Health (total transformation budget €2.8 billion). We may anticipate a shift within the regions towards more population health management. This mirrors developments seen in England, where since 2019 primary care networks (PCNs) (covering around 30,000–45,000 patients) were formed to coordinate the collaboration between GP practices and community, mental health, social care, pharmacy, hospital and voluntary services. The implementation of PCNs involved an investment of £4.5 billion over five years to enhance the sustainability of the healthcare system in terms of workforce, finance, and the promotion of disease prevention [44, 45]. In England, various entities are in place to improve the integration of care: (1) the integrated care systems (ICSs) (covering 500,000–3,000,000 people) that align resources, integrate resources across settings, and coordinate the entire healthcare system in a certain region [44], (2) the integrated care boards (ICBs) and integrated care partnerships (ICPs) which recently replaced clinical commissioning groups which previously organized and purchased the healthcare services according to its patient population needs. Now, ICBs and ICPs took over these tasks and operate within the ICS structure, holding direct accountability to NHS England for financial and performance outcomes, and (3) the primary care networks (PCNs) consist of groups of GP-practices working together to deliver integrated care in the community. Clear outcomes of the reforms are not available yet, but the national health system (NHS) reports different case studies, which show that the PCNs have a positive contribution for patients and practitioners [46].

The practicalities of contracting the expanded service package and alternative payment model for the PCC should not be underestimated. The PCC is responsible for the coordination and content of the care package and negotiates with the health insurer about one contract for the bundled payment. Subsequently, the PCC subcontracts the healthcare providers who provide the services in the bundle [1]. PCCs shared their fear for additional administrative workload if different (financial) agreements have to be made with many different providers. However, we have shown that it is possible to expand the care package with acceptable risks, so the PCC could take up a more central role in the coordination of wider chronic disease management.

## Conclusion

To conclude, we characterized the OPTIMA FORMA target population by distinguishing six groups from the heterogeneous population with DM2, CVRM and/or COPD which is currently receiving care from one or multiple SDMPs. We were able to reliably predict upfront to which of six clusters a patient belongs and use this information to make risk-adjusted predictions of healthcare expenditure at the PCC level. Using ‘relevance of a service for patient-centred care’ and ‘financial risk to the PCC’ as criteria, we were able to operationalize the person-centred bundled-payment model by constructing different care packages. We showed that mainly the size of the PCC and the content of the care package influenced the predicted losses or profits for the PCC.

## Supplementary Information

Below is the link to the electronic supplementary material.

Supplementary file1 (DOCX 309 kb)

## Data Availability

The dataset generated and analyzed during the current study might be available from the corresponding author on request.

## Footnotes

1 Other sectors contained alternative healthcare and other remaining costs not belonging to any of the other sectors.

2 The 12 chronic diseases are: acid related disorders, bone diseases, CVRM, diabetes mellitus, glaucoma, gout, hyperlipidemia, pain, psychological disorders, respiratory illnesses, rheumatoid, and thyroid disorders.

3 The 13 medical specialists are: ophthalmology, ear, nose, throat specialist, surgeon, orthopedics, urology, dermatology, internal medicine, gastroenterology, cardiology, pulmonary illness, rheumatology, neurology, and geriatrics.
