# Supplementary material for: Characterizing a heterogeneous chronic patient population for redesigning person-centred bundled payment models using risk-mitigating measures
Source: Eur J Health Econ. 2025 Mar 15;26(7):1249–62. doi: 10.1007/s10198-025-01762-x (PMC12432071; doi:10.1007/s10198-025-01762-x)
Supplement: Supplementary file 1 — Supplementary file1 (DOCX 309 kb) [file 10198_2025_1762_MOESM1_ESM.docx]

**Supplementary files**

**List of tables**

[Table A. List of variables included in and excluded from the cluster analysis per healthcare sector. 23](#_Toc181634455)

[Table B. Mean total costs of care by number of chronic diseases. 26](#_Toc181634456)

[Table C. Stability of the 5 clusters. 27](#_Toc181634457)

[Table D. Stability of the 6 clusters. 28](#_Toc181634458)

[Table E. Stability of the 8 clusters. 28](#_Toc181634459)

[Table F. Most important characteristics of the different clusters. 29](#_Toc181634460)

[Table G. Percentage of patients who uses medical care from a certain specialism. 29](#_Toc181634461)

[Table H. Healthcare expenditures per health sector. 30](#_Toc181634462)

[Table I. Observed event rate development set and validation set and calibration-in-the-large of validation set of PCC 1 (small PCC). 31](#_Toc181634463)

[Table J. Observed event rate development set and validation set and calibration-in-the-large of validation set of PCC 2 (medium PCC). 31](#_Toc181634464)

[Table K. Observed event rate development set and validation set and calibration-in-the-large of validation set of PCC 3 (large PCC). 32](#_Toc181634465)

[Table L. Polytomous discrimination indexes. 32](#_Toc181634466)

[Table M. Sensitivity and specificity the seven MNL models to predict cluster probabilities. 33](#_Toc181634467)

[Table N. Relevance of extending the single disease management programmes. 34](#_Toc181634468)

[Table O. Mean predicted profits or losses per primary care cooperative for the extension of the currently used SDMPs. 37](#_Toc181634469)

**List of figures**

[Figure A. Variation between patients in a cluster (WSS) and the proportional reduction of error (PRE) for one to twenty clusters. 25](#_Toc158993496)

## Appendix A List of cluster variables

From all variables in our dataset, we created a list (table A, column 2) of variables which were included in the cluster analysis. Some of these variables were a combination of variables from the dataset (column 3: combined variables). In table A, we also show all the variables that were part of the dataset but were not included in the cluster analysis (column 4).

Table A. List of variables included in and excluded from the cluster analysis per healthcare sector.

| **Healthcare sector** | **Variables included in cluster analysis** | **Combined variables** | **Variables not in cluster analysis** |
| --- | --- | --- | --- |
| **Primary care** | **Regular consultation** |  | Capitation payment |
|  | **Visits** |  | Capitation payment with additional payment for living in a deprived area |
|  | **Surgery in the GP practice** |  | Capitation payment for the mental nurse |
|  | **Diagnostics of urinary tract infection** |  | Other services in the GP practice |
|  | **Blood sugar determination** |  | Organization of integrated care |
|  | **Stop smoking modules** |  | Innovation and reward payment |
|  | **Mental health in the GP practice** | Consultations + visits of primary care psychologist |  |
|  | **Combined services in the GP practice** | Primary care stay + intensive care in the evening, night, or weekend + intensive care during the day + evening, night, and weekend services |  |
|  | **SDMP CVRM** | tariff CVRM + SDMP CVRM |  |
|  | **SDMP DM2** | SDMP DM2 and CVRM + SDMP DM2 |  |
|  | **SDMP COPD** | SDMP asthma + SDMP COPD |  |
| **Paramedical care** | **Physiotherapy** | Therapeutic injection + physiotherapy additional package + physiotherapy basic package |  |
|  | **Other paramedical care** | **Additional package**: Exercise therapy + occupational therapy + diet advice + skin care + footcare +  **Basic package**: Exercise therapy + speech therapy + occupational therapy + diet advice |  |
| **Pharmaceutical care** | **Acid related disorders** |  | Cancer |
|  | **Bone diseases (osteoporosis)** |  | HIV |
|  | **Cardiovascular diseases (incl. hypertension)** |  | Intestinal inflammatory diseases |
|  | **Epilepsy** |  | Iron deficiency anaemia |
|  | **Glaucoma** |  | Migraines |
|  | **Psychological Psychoses** | Psychological disorders (sleep disorder, depression) and Psychoses | Tuberculosis |
|  | **Dementia Parkinson** | Dementia and Parkinson’s disease |  |
|  | **Hyperlipidaemia** |  |  |
|  | **Pain** |  |  |
|  | **Respiratory illness (asthma/COPD)** |  |  |
|  | **Rheumatologic conditions** | Gout, Hyperuricemia and Rheumatologic conditions |  |
|  | **Thyroid disorders** |  |  |
|  |  |  |  |
| **Medical specialist** | **Ophthalmology** |  | Plastic surgeon |
|  | **Ear, nose, throat specialist** |  | obstetrics |
|  | **Surgery** |  | paediatrics |
|  | **Orthopedics** |  | Allergology |
|  | **Urology** |  | Psychiatrist |
|  | **Dermatology** |  | Radiotherapy |
|  | **Internal medicine** |  | Radiology |
|  | **Gastroenterology** |  | Clinical genetics |
|  | **Cardio** | Cardiology and Cardiopulmonary | Audiology |
|  | **Pulmonary disease** |  | Expensive medicine |
|  | **Neuro** | Neurosurgeon and Neurology | Medical specialist care at home |
|  | **Geriatric** |  | Other products |
|  | **Rheumatology** |  |  |
|  | **Rehabilitation** |  |  |
|  | **Anesthesiology** |  |  |
|  | **Diagnostics** |  |  |
| **Mental health care** | **Basic mental health care** | Basic mental health care short + Basic mental health care medium + Basic mental health care intensive + Basic mental health care chronic + Basic mental health care incomplete |  |
|  | **Specialist mental health care** | Diagnostics + crisis + attention + pervasive disorder+ alcohol addiction + addiction + classification + depression + bipolar disorder + anxiety disorder + diverse + personality disorder + short treatment + dementia + somatoform disorder + eating disorder + stay + electrotherapy + methadone + stay without night + interpreter |  |
| **First line stays, Geriatric rehabilitation care, and district nursing** | **First line stays, Geriatric rehabilitation care, and district nursing** | ELV low + ELV high + ELV palliative + GRZ, personal care + public care + nursing + public nursing + specialist nursing + advice and instruction + indication + personal budget formal care + personal budget informal care + personal budget formal nursing + personal budget informal nursing + personal budget care and nursing + pharma telecare + complex wound care + experiment district nursing + integrated district nursing + short care after hospital admission + care for elderly and chronically ill, shorter than 3 months + care for elderly and chronically ill, longer than 3 months + care for elderly and chronically ill, longer than 3 months (mentally) + Prevention + Palliative care | Reward district nursing |
| **Medical aids (basic care package and additional care package)** | **Orthoses and orthopaedic footwear** | Orthoses not specified + orthoses leg/foot + orthopedic footwear not specified + Orthoses + Orthopedic footwear + footwear | Bandages (basic care package and additional care package) |
|  | **Problems with urinating** | Incontinence materials + medical aids for urinating | Hearing aids (basic care package and additional care package) |
|  | **medical aids for diabetes** |  | Glasses |
|  | **Support for blood and lymph** |  | Visual aids (basic care package and additional care package) |
|  | **Breathing medical aids** |  | Medical aids to differ the posture |
|  | **Elements for the house** |  | Wigs |
|  |  |  | Personal alarm system |
|  |  |  | Stoma |
|  |  |  | Protheses and wigs |
|  |  |  | Communication aids |
|  |  |  | Aids to increase mobility of the person |
|  |  |  | Nutrition aids |
|  |  |  | Aids related to a treatment |
|  |  |  | Aids to improve the arm, hand, or finger function |
|  |  |  | Aids for home dialyses |
| **Transport** | **Transport** | Ambulance + other transport |  |
| **Other** |  |  | Alternative healthcare |
|  |  |  | Other healthcare |

## Appendix B. Mean total costs of care by the number of chronic diseases per patient.

Table B. Mean total costs of care by number of chronic diseases.

| **Number of chronic diseases** | **N** | **Mean total costs** | **SD** | **Median** | **Range 25%-75%** |
| --- | --- | --- | --- | --- | --- |
| **1** | 8,585 | € 2,808 | €8,991 | €894 | €475 - €2,089 |
| **2** | 11,441 | € 3,758 | €8,664 | €1,390 | €734 - €3,061 |
| **3** | 10,481 | € 5,691 | €10,574 | €2,259 | €1,211 - €5,135 |
| **4** | 7,109 | € 8,084 | €12,240 | €3,613 | €1,884 - €8,656 |
| **5 or more** | 5,711 | € 12,630 | €16,247 | €6,451 | €3,292 - €15,778 |

## Appendix C. Determining the optimal number of clusters.

We estimated the within sum square (WSS) and the proportional reduction of error (PRE) to define the optimal number of clusters. The WSS is diminishing relatively quicker for five, six, and eight clusters. For both six and eight clusters, less reduction is observed of WSS by adding an additional cluster. The PRE, the proportional reduction of the WSS, shows an unstable pattern above eight clusters (the PRE is sometimes 0 or even below 0). The PRE is relatively high for five, six and eight clusters.

Figure A. Variation between patients in a cluster (WSS) and the proportional reduction of error (PRE) for one to twenty clusters.


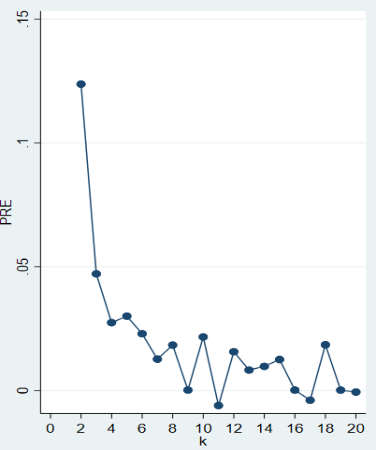

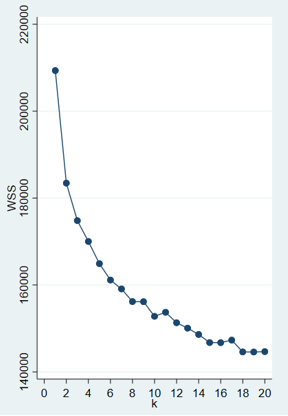


For these three numbers of clusters, we compared the base case with four different seed values. Cluster analysis is inherently sensitive to variations in partitioning due to random starting values. To address this, we assessed the stability of cluster allocations by using different seed values. Tables C to E show the results on stability. The higher the percentages, the more stable the clusters are. The six-cluster solution is more stable compared to the stability of five- and eight-cluster solutions. Due to this criterion the optimal number of clusters is six.

Table C. Stability of the 5 clusters.

| **Original cluster** | **Seed value 1** | **Seed value 2** | **Seed value 3** | **Seed value 4** |
| --- | --- | --- | --- | --- |
| 1 | 99% cluster 5 | 99% cluster 3 | 99% cluster 5 | 99% cluster 2 |
| 2 | 94% cluster 1 | 94% cluster 5 | 71% cluster 3 | 94% cluster 1 |
| 3 | 87% cluster 3 | 87% cluster 2 | 100% cluster 1 | 87% cluster 4 |
| 4 | 80% cluster 1 | 80% cluster 5 | 80% cluster 3 | 80% cluster 1 |
| 5 | 52% cluster 2 | 52% cluster 4 | 95% cluster 2 | 52% cluster 5 |

Table D. Stability of the 6 clusters.

| **Original cluster** | **Seed value 1** | **Seed value 2** | **Seed value 3** | **Seed value 4** |
| --- | --- | --- | --- | --- |
| 1 | 100% cluster 5 | 88% cluster 2 | 83% cluster 6 | 74% cluster 6 |
| 2 | 100% cluster 1 | 76% cluster 2 | 75% cluster 6 | 74% cluster 6 |
| 3 | 100% cluster 4 | 98% cluster 3 | 99% cluster 4 | 93% cluster 2 |
| 4 | 100% cluster 6 | 99% cluster 6 | 99.9% cluster 5 | 99,9% cluster 3 |
| 5 | 100% cluster 3 | 99% cluster 5 | 99% cluster 2 | 99% cluster 1 |
| 6 | 100% cluster 2 | 93% cluster 4 | 92% cluster 1 | 91% cluster 6 |

Table E. Stability of the 8 clusters.

| **Original cluster** | **Seed value 1** | **Seed value 2** | **Seed value 3** | **Seed value 4** |
| --- | --- | --- | --- | --- |
| 1 | 51% cluster 2 | 81% cluster 7 | 81% cluster 7 | 91% cluster 3 |
| 2 | 72% cluster 7 | 72% cluster 8 | 90% cluster 1 | 86% cluster 7 |
| 3 | 97% cluster 8 | 96% cluster 3 | 97% cluster 8 | 97% cluster 1 |
| 4 | 59% cluster 6 | 54% cluster 4 | 28% cluster 3 | 91% cluster 8 |
| 5 | 96% cluster 1 | 100% cluster 1 | 90% cluster 4 | 98% cluster 4 |
| 6 | 56% cluster 2 | 98% cluster 6 | 82% cluster 2 | 98% cluster 2 |
| 7 | 76% cluster 7 | 72% cluster 8 | 99% cluster 5 | 99% cluster 5 |
| 8 | 80% cluster 5 | 100% cluster 5 | 51% cluster 4 | 91% cluster 4, |

## Appendix D. Characteristics of patients in the different clusters.

Tables F, G and H provide information about the six clusters.

Table F. Most important characteristics of the different clusters.

| **Clusters** | **Cluster 1** | **Cluster 2** | **Cluster 3** | **Cluster 4** | **Cluster5** | **Cluster 6** |
| --- | --- | --- | --- | --- | --- | --- |
| **Number of patients n (%)** | 15667 (36%) | 7871 (18%) | 1296 (3%) | 11397 (26%) | 4185 (10%) | 2911 (7%) |
| **Female** | 55% | 58% | 51% | 48% | 61% | 66% |
| **Average age (Interquartile)** | 65 (15) | 68 (15) | 63 (16) | 67 (16) | 75 (14) | 76 (15) |
| **Died in 2017** | 0% | 0% | 1% | 1% | **10%** | **12%** |
| **Number of chronic diseases** | | | | | | |
| 1 | **42%** | 0% | **43%** | 8% | 4% | 14% |
| 2 | **43%** | 18% | 29% | 18% | 7% | 24% |
| 3+ | 15% | **82%** | 28% | **75%** | **90%** | **62%** |
| **Declared SDMP** | | | | | | |
| Only in DM2 | 0% | 0% | 0% | **96%** | **91%** | 0% |
| Only in CVR | **96%** | **89%** | 0% | 0% | 0% | **84%** |
| Only in COPD | 0% | 2% | **81%** | 0% | 1% | 4% |
| DM2 + CVR | 1% | 1% | 0% | 1% | 1% | 1% |
| DM2 + COPD | 0% | 0% | 3% | 3% | **7%** | 0% |
| CVR + COPD | 3% | 7% | **15%** | 0% | 0% | 10% |
| **Most common chronic diseases based on medication use** | | | | | | |
| Acid related disorders | 0% | **96%** | 17% | 35% | 73% | 52% |
| Bone diseases (osteoporosis) | 1% | 4% | 3% | 1% | 6% | **10%** |
| Cardiovascular diseases (incl. hypertension) | 74% | **87%** | 19% | 74% | **86%** | 81% |
| Diabetes mellitus | 0% | 0% | 1% | **74%** | **70%** | 0% |
| Epilepsy | 1% | 4% | 2% | 2% | 7% | **8%** |
| Hyperlipidemia | 43% | 63% | 9% | **73%** | 65% | 38% |
| Pain | 1% | 7% | 3% | 3% | **16%** | **15%** |
| Psychological disorders (sleep disorder, depression) | 6% | 16% | 13% | 10% | **21%** | 19% |
| Respiratory illness (asthma/COPD) | 6% | 20% | **74%** | 9% | 28% | 32% |
| Rheumatologic conditions | 2% | **13%** | 5% | 4% | 8% | 7% |
| Thyroid disorders | 5% | 8% | 5% | 7% | **10%** | 9% |

Table G. Percentage of patients who uses medical care from a certain specialism.

| **Most used specialisms** | **Cluster 1** | **Cluster 2** | **Cluster 3** | **Cluster 4** | **Cluster5** | **Cluster 6** |
| --- | --- | --- | --- | --- | --- | --- |
| Ophthalmology | 11% | 17% | 8% | 20% | **32%** | 21% |
| Ear, nose, throat specialist | 5% | 8% | 7% | 5% | 10% | **11%** |
| Surgery | 8% | 16% | 10% | 9% | 33% | **36%** |
| Orthopedics | 7% | 13% | 6% | 7% | 15% | **17%** |
| Urology | 4% | 8% | 6% | 5% | **15%** | **15%** |
| Dermatology | 9% | 14% | 8% | 9% | **17%** | 16% |
| Internal medicine | 6% | 12% | 7% | 9% | **43%** | 35% |
| Gastroenterology | 4% | 11% | 7% | 6% | 16% | **17%** |
| Cardiology | 7% | 21% | 8% | 15% | **44%** | 36% |
| Pulmonary disease | 3% | 10% | 11% | 5% | 26% | **29%** |
| Rheumatology | 2% | **8%** | 2% | 3% | 7% | **8%** |
| Rehabilitation | 1% | 2% | 1% | 1% | 6% | **8%** |
| Neurology | 5% | 12% | 6% | 5% | 22% | **28%** |
| Geriatric | 1% | 1% | 1% | 1% | 14% | **17%** |
| Anesthesiology | 1% | 4% | 2% | 2% | 7% | **8%** |
| Expensive medicines | 2% | 4% | 3% | 2% | 7% | **9%** |
| Diagnostics | 87% | 91% | 60% | **95%** | **95%** | 87% |
| Medical specialist care at home | 0% | 1% | 1% | 0% | **8%** | 7% |
| Other products | 7% | 11% | 6% | 11% | **34%** | 30% |
| **Number of specialists** | | | | | | |
| 0 | **51%** | 26% | **47%** | 39% | 8% | 7% |
| 1 | 29% | 27% | 28% | **31%** | 12% | 15% |
| 2 | 13% | **22%** | 15% | 17% | 19% | 18% |
| 3+ | 7% | 25% | 10% | 12% | **61%** | **60%** |

Table H. Healthcare expenditures per health sector.

| **Sector** | **Cluster 1**  **avg (Inter**  **quartile)** | **Cluster 2 avg (Inter**  **quartile** | **Cluster 3**  **avg (Inter**  **quartile)** | **Cluster 4**  **avg (Inter**  **quartile)** | **Cluster 5 avg**  **(Inter**  **quartile)** | **Cluster 6**  **avg (Inter**  **quartile)** |
| --- | --- | --- | --- | --- | --- | --- |
| General practitioner (S1) | €144 (64) | €189 (93) | €160 (82) | €161 (74) | **€313 (163)** | **€329 (183)** |
| General practitioner (S2) | €153 (24) | €168 (24) | €255 (9) | **€375 (42)** | **€363 (42)** | €160 (24) |
| General practitioner (other) | €15 (4) | €29 (30) | €23 (13) | €19 (7) | €113 (152) | €120 (174) |
| Paramedical (from additional health insurance package) | €98 (107) | €187 (286) | €91 (77) | €110 (121) | **€246 (384)** | **€281 (459)** |
| Paramedical (from basic health insurance package) | €32 (0) | €99 (0) | €61 (0) | €43 (0) | **€238 (63)** | **€313 (202)** |
| Stop smoking modules | €2 (0) | €3 (0) | **€12 (0)** | €1 (0) | €2 (0) | €3 (0) |
| Basic mental care | €8 (0) | €14 (0) | €22 (0) | €8 (0) | €13 (0) | €9 (0) |
| Specialistic mental care | €124 (0) | €252 (0) | €356 (0) | €135 (0) | €450 (0) | **€626 (0)** |
| Primary care stay | €7 (0) | €3 (0) | €1 (0) | €10 (0) | €280 (0) | **€444 (0)** |
| Pharmaceuticals | €300 (259) | €761 (743) | €930 (786) | €752 (746) | **€1843 (1443)** | €1375 (1239) |
| Rehabilitation care | €3 (0) | €6 (0) | €0 | €3 (0) | €560 (0) | **€813 (0)** |
| Medical aids (from additional health insurance package) | €17 (0) | €24 (0) | €17 (0) | €18 (0) | €22 (0) | €23 (0) |
| Medical aids (from basic health insurance package) | €75 (0) | €188 (105) | €119 (24) | €165 (142) | **€897 (891)** | €782 (771) |
| Medical specialist care (DRGs) | €877 (702) | €2313 (2202) | €1177 (1018) | €1174 (1025) | €7208 (8212) | **€7852 (9448)** |
| Medical specialist care (other) | €226 (65) | €494 (139) | €243 (102) | €283 (122) | €1551 (504) | **€1891 (511)** |
| Transport to hospital | €14 (0) | €50 (0) | €25 (0) | €16 (0) | €380 (713) | **€452 (725)** |
| District nursing | €75 (0) | €276 (0) | €169 (0) | €207 (0) | **€4549 (6032)** | **€4406 (5639)** |
| Other sectors | €20 (0) | €30 (0) | €23 (0) | €21 (0) | €70 (0) | €72 (0) |
| Total | **€2175 (1559)** | **€5055 (3993)** | **€3693 (2595)** | **€3513 (2550)** | **€19050 (€17472)** | **€19903 (18256)** |

## Appendix E. Performance of seven prediction models

In order to use these clusters in practice we have to be able to predict upfront which patient belongs to which cluster and estimate the proportion of patients belonging to a cluster.

We estimated 7 different MNL-models, model 1 incorporated least information and model 7 most information. We expected that model 1 would perform worst and model 7 best in predicting which patient belongs to which cluster, so we are mostly interested in finding the optimal model, i.e., the model with good performance when using less variables.

We are mainly interested in how well the models are calibrated, since we will use them to predict costs, and therefore we need to know how well the models predict the overall proportion of patients per cluster per PCC. Therefore, we first estimated the calibration-in-the-large. Tables I, J and K show that the predicted probabilities of cluster membership of MNL models 3, 5 and 7 deviated the least from the observed probability of patients per cluster and for models 2 and 6 the most. Tables I and K show that for PCCs 1 and 3 the models 1, 2, 4, and 6 overpredict the probability of being in cluster 1 and underestimate the probability of being in cluster 2. Table J shows a similar pattern for PCC 2 but the other way around. All models predict the proportion of patients in cluster 3 well. Also, clusters 4, 5 and 6 only show little variation between the observed probabilities and the predicted probabilities of all models.

Table I. Observed event rate development set and validation set and calibration-in-the-large of validation set of PCC 1 (small PCC).

|  | **Observed event rate development data (n=43327)** | **Observed event rate validation data (n=1514)** | **Expected event rate validation data** | | | | | | |
| --- | --- | --- | --- | --- | --- | --- | --- | --- | --- |
|  |  |  | **Model 1** | **Model 2** | **Model 3** | **Model 4** | **Model 5** | **Model 6** | **Model 7** |
| **Cluster 1** | 36.16% | 30.45% | 32.32% | 32.55% | 30.63% | 31.52% | 30.78% | 31.77% | 30.44% |
| **Cluster 2** | 18.17% | 17.44% | 15.77% | 15.76% | 17.34% | 15.96% | 17.61% | 16.12% | 17.63% |
| **Cluster 3** | 2.99% | 7.00% | 6.66% | 6.84% | 6.70% | 6.91% | 6.86% | 7.01% | 6.86% |
| **Cluster 4** | 26.30% | 31.18% | 30.98% | 31.26% | 30.90% | 31.76% | 31.15% | 31.83% | 31.46% |
| **Cluster 5** | 9.66% | 8.52% | 8.80% | 8.46% | 8.88% | 7.99% | 8.59% | 7.90% | 8.27% |
| **Cluster 6** | 6.72% | 5.42% | 5.44% | 5.09% | 5.53% | 5.84% | 5.02% | 5.35% | 5.32% |
| **Total calibration-in-the-large** | | | 4.38 | 4.41 | 1.33 | 4.17 | 1.14 | 4.32 | 0.97 |

Table J. Observed event rate development set and validation set and calibration-in-the-large of validation set of PCC 2 (medium PCC).

|  | **Observed event rate development data (n=43327)** | **Observed event rate validation data (n=14740)** | **Expected event rate validation data** | | | | | | |
| --- | --- | --- | --- | --- | --- | --- | --- | --- | --- |
|  |  |  | **Model 1** | **Model 2** | **Model 3** | **Model 4** | **Model 5** | **Model 6** | **Model 7** |
| **Cluster 1** | 33.68% | 40.96% | 38.35% | 38.28% | 40.86% | 39.08% | 40.80% | 38.99% | 40.95% |
| **Cluster 2** | 19.03% | 16.49% | 18.75% | 18.74% | 16.42% | 18.41% | 16.31% | 18.34% | 16.29% |
| **Cluster 3** | 2.85% | 3.27% | 3.36% | 3.34% | 3.28% | 3.25% | 3.28% | 3.24% | 3.29% |
| **Cluster 4** | 27.84% | 23.32% | 23.30% | 23.05% | 23.41% | 23.25% | 23.22% | 23.04% | 23.16% |
| **Cluster 5** | 9.76% | 9.46% | 9.43% | 9.69% | 9.36% | 9.51% | 9.56% | 9.72% | 9.60% |
| **Cluster 6** | 6.84% | 6.49% | 6.79% | 6.89% | 6.67% | 6.48% | 6.83% | 6.65% | 6.71% |
| **Total calibration-in-the-large** | | | 5.31 | 5.90 | 0.55 | 4.05 | 0.99 | 4.65 | 0.85 |

Table K. Observed event rate development set and validation set and calibration-in-the-large of validation set of PCC 3 (large PCC).

|  | **Observed event rate development data (n=43327)** | **Observed event rate validation data (n=27073)** | **Expected event rate validation data** | | | | | | |
| --- | --- | --- | --- | --- | --- | --- | --- | --- | --- |
|  |  |  | **Model 1** | **Model 2** | **Model 3** | **Model 4** | **Model 5** | **Model 6** | **Model 7** |
| **Cluster 1** | 33.68% | 33.86% | 35.19% | 35.21% | 33.91% | 34.83% | 33.93% | 34.86% | 33.87% |
| **Cluster 2** | 19.03% | 19.12% | 17.97% | 17.98% | 19.16% | 18.15% | 19.20% | 18.18% | 19.22% |
| **Cluster 3** | 2.85% | 2.62% | 2.57% | 2.57% | 2.62% | 2.62% | 2.62% | 3.24% | 2.61% |
| **Cluster 4** | 27.84% | 27.66% | 27.68% | 27.79% | 27.62% | 27.66% | 27.71% | 23.04% | 27.73% |
| **Cluster 5** | 9.76% | 9.83% | 9.82% | 9.70% | 9.86% | 9.83% | 9.77% | 9.72% | 9.77% |
| **Cluster 6** | 6.84% | 6.91% | 6.75% | 6.71% | 6.81% | 6.90% | 6.75% | 6.83% | 6.80% |
| **Total calibration-in-the-large** | | | 2.72 | 3.00 | 0.26 | 1.95 | 0.42 | 7.37 | 0.36 |

We decided that the originally developed models fit well enough for each local setting and therefore, we do not need to recalibrate the model for the local settings. However, in case it is needed Van Calster et al [29] explain how to recalibrate a prediction model in detail.

Secondly, we measured the performance of the different models on the polytomous discrimination indexes (PDIs). Table L shows the overall PDIs for the development dataset and for each PCC. The overall polytomous discrimination indexes (PDIs) are highest for models 5 and 7, which almost achieve perfect discrimination (PDI=1). Model 1 performs worst in discriminating between cluster-probabilities. The performance of model 2 improves substantially compared to model 1.

Table L. Polytomous discrimination indexes.

|  | **Polytomous discrimination index** | | | | | | |
| --- | --- | --- | --- | --- | --- | --- | --- |
|  | **Model 1** | **Model 2** | **Model 3** | **Model 4** | **Model 5** | **Model 6** | **Model 7** |
| **Development set** | 0.622 | 0.726 | 0.834 | 0.765 | 0.911 | 0.820 | 0.958 |
| **Small (n=1514)** | 0.657 | 0.742 | 0.847 | 0.766 | 0.913 | 0.814 | 0.962 |
| **Medium (n=14740)** | 0.623 | 0.731 | 0.832 | 0.761 | 0.914 | 0.820 | 0.957 |
| **Large (n=27073)** | 0.618 | 0.722 | 0.833 | 0.766 | 0.908 | 0.820 | 0.958 |

Although we are more interested in predicting the right proportions of patients per cluster than in predicting the right cluster for an individual patient, we did investigate the latter because it an important measure to check if there is a risk for risk selection. Table M provides more detail on the classification estimates. It shows that specificity was generally higher than sensitivity. Only model 5 and model 7 correctly identify more than 60% of patients in all six clusters. The other models generally performed less well, especially in predicting cluster-probabilities for clusters 2, 5 and 6. Although model 6 had a higher sensitivity than model 5 for clusters 5 and 6, the sensitivity for cluster 2 was substantially lower. Data on chronic diseases improved the prediction of cluster-probabilities for clusters 2 and 5, while the addition of medical specialists and district nursing improved this prediction for clusters 5 and 6.

Overall, model 5 is considered the optimal performing model. We decided to use the predictions from model 5, the optimal model, to predict the “risk” of implementing more extensive care packages for patients with chronic diseases.

Table M. Sensitivity and specificity the seven MNL models to predict cluster-probabilities.

|  | **Cluster 1** | | | | **Cluster 2** | | | | **Cluster 3** | | | |
| --- | --- | --- | --- | --- | --- | --- | --- | --- | --- | --- | --- | --- |
| 1. **Simple** | **D** | **PCC1** | **PCC2** | **PCC3** | **D** | **PCC1** | **PCC2** | **PCC3** | **D** | **PCC1** | **PCC2** | **PCC3** |
| Sensitivity | **96%** | 97% | 97% | 96% | **6%** | 8% | 6% | 7% | **81%** | 91% | 81% | 80% |
| Specificity | **66%** | 72% | 66% | 65% | **98%** | 98% | 98% | 98% | **99%** | 99% | 99% | 99% |
| 1. **District nursing model** | | | | | | | | | | | | |
| Sensitivity | **95%** | 97% | 95% | 95% | **6%** | 8% | 6% | 7% | **79%** | 90% | 79% | 78% |
| Specificity | **72%** | 76% | 73% | 72% | **98%** | 99% | 98% | 98% | **99%** | 99% | 99% | 100% |
| 1. **Chronic medication model** | | | | | | | | | | | | |
| Sensitivity | **99%** | 99% | 99% | 99% | **94%** | 95% | 93% | 94% | **91%** | 92% | 91% | 90% |
| Specificity | **94%** | 96% | 94% | 95% | **97%** | 97% | 97% | 96% | **100%** | 100% | 100% | 100% |
| 1. **Specialist care model** | | | | | | | | | | | | |
| Sensitivity | **91%** | 90% | 92% | 89% | **26%** | 24% | 25% | 27% | **79%** | 88% | 78% | 79% |
| Specificity | **78%** | 81% | 78% | 78% | **94%** | 94% | 94% | 93% | **100%** | 99% | 99% | 100% |
| 1. **District nursing + chronic medication model** | | | | | | | | | | | | |
| Sensitivity | **98%** | 99% | 98% | 98% | **91%** | 94% | 90% | 92% | **91%** | 93% | 92% | 91% |
| Specificity | **96%** | 97% | 96% | 97% | **99%** | 99% | 99% | 98% | **100%** | 100% | 100% | 100% |
| 1. **District nursing + specialist care model** | | | | | | | | | | | | |
| Sensitivity | **90%** | 89% | 91% | 89% | **28%** | 25% | 27% | 29% | **80%** | 89% | 78% | 79% |
| Specificity | **80%** | 82% | 80% | 80% | **95%** | 95% | 95% | 94% | **100%** | 99% | 99% | 100% |
| 1. **District nursing + chronic medication + specialist care model** | | | | | | | | | | | | |
| Sensitivity | **98%** | 98% | 99% | 98% | **93%** | 95% | 92% | 93% | **92%** | 94% | 93% | 92% |
| Specificity | **97%** | 98% | 97% | 97% | **99%** | 99% | 99% | 99% | **100%** | 100% | 100% | 100% |

**D=development dataset, C1= primary care cooperative 1, C2= primary care cooperative 2, and C3 is primary care cooperative 3.*

|  | **Cluster 4** | | | | **Cluster 5** | | | | **Cluster 6** | | | |
| --- | --- | --- | --- | --- | --- | --- | --- | --- | --- | --- | --- | --- |
| 1. **Simple** | **D** | **C1** | **C2** | **C3** | **D** | **C1** | **C2** | **C3** | **D** | **C1** | **C2** | **C3** |
| Sensitivity | **95%** | 97% | 93% | 95% | **21%** | 9% | 26% | 19% | **12%** | 12% | 12% | 12% |
| Specificity | **90%** | 89% | 91% | 89% | **99%** | 99% | 98% | 99% | **99%** | 100% | 99% | 99% |
| 1. **District nursing model** | | | | | | | | | | | | |
| Sensitivity | **95%** | 97% | 94% | 95% | **56%** | 49% | 61% | 53% | **67%** | 63% | 69% | 66% |
| Specificity | **94%** | 94% | 95% | 94% | **99%** | 99% | 98% | 99% | **98%** | 99% | 97% | 98% |
| 1. **Chronic medication model** | | | | | | | | | | | | |
| Sensitivity | **92%** | 96% | 92% | 92% | **44%** | 45% | 47% | 43% | **17%** | 17% | 18% | 16% |
| Specificity | **93%** | 93% | 94% | 92% | **98%** | 98% | 98% | 98% | **99%** | 99% | 99% | 99% |
| 1. **Specialist care model** | | | | | | | | | | | | |
| Sensitivity | **93%** | 95% | 92% | 93% | **58%** | 50% | 59% | 59% | **44%** | 46% | 44% | 43% |
| Specificity | **95%** | 94% | 95% | 94% | **96%** | 99% | 98% | 98% | **98%** | 98% | 98% | 98% |
| 1. **District nursing + chronic medication model** | | | | | | | | | | | | |
| Sensitivity | **94%** | 96% | 94% | 94% | **63%** | 63% | 68% | 61% | **60%** | 55% | 61% | 59% |
| Specificity | **95%** | 96% | 96% | 95% | **98%** | 99% | 98% | 98% | **98%** | 99% | 98% | 98% |
| 1. **District nursing + specialist care model** | | | | | | | | | | | | |
| Sensitivity | **94%** | 95% | 92% | 94% | **73%** | 64% | 76% | 72% | **70%** | 68% | 70% | 69% |
| Specificity | **96%** | 95% | 97% | 96% | **98%** | 98% | 98% | 98% | **98%** | 99% | 98% | 98% |
| 1. **District nursing + chronic medication + specialist care model** | | | | | | | | | | | | |
| Sensitivity | **95%** | 96% | 94% | 95% | **77%** | 74% | 80% | 76% | **67%** | 73% | 68% | 67% |
| Specificity | **97%** | 97% | 98% | 97% | **98%** | 99% | 98% | 99% | **98%** | 99% | 98% | 99% |

## Appendix F. Relevance of care in the person-centred bundled payment

Table N explains the relevance of all different types of care. The relevance is determined by the percentage of people who use a certain type of care, whether the type of care was mentioned in the standard of care, and whether a pattern of care could be found due to cluster analysis. Relevance is *high* when more than 5% use a certain type of care and it is mentioned in the standard of care, as well as when a certain type of care is used more by a certain cluster in combination with when it is mentioned in the standard of care. The relevance was also determined as high when all standards of care named the certain type of care. The relevance is *medium* when only one of the two conditions hold. The relevance is low if none of the criteria hold or when it is not possible to include the type of care because the content is unclear.

Table N. Relevance of extending the single disease management programmes.

| Extend the current SDMPs with … | % Usage | Arguments based on the standard of care* | Relevance |
| --- | --- | --- | --- |
| All general practitioners care | 99% | To avoid confusion about which care is part and which care is not part of the bundle payment. All services should be included in the bundled payment. | High |
| Physiotherapy | 34% | Physiotherapy is partly included in the current bundle, but still 34% of the patients uses care outside the bundled payment. It could be beneficial to include physiotherapy for prevention. | High |
| Foot therapy/ pedicure | 8% | Foot therapy is already partly in the current bundle. When the bundles are combined all foot therapy should be included in the bundle. Foot therapy is currently not part of the care programs for patients with CVRM or COPD, which will change. | High |
| Ergotherapy | 3% | Named in the standard of care for patients with COPD and used more by people in clusters 5 and 6. | High |
| Dietician | 3% | A part is already included in the bundle for DM2. Dietary care could also be preventive for progression to other (chronic) diseases. | High |
| Practice therapy | 2% | Named in the standard of care for patients with CVRM and used more by people in clusters 5 and 6. | High |
| Speech therapy | 1% | - | Low |
| Skin therapy | 0,3% | - | Low |
| Stop smoking modules | 1% | Named in the standard of care for patients with DM2, CVRM and COPD as prevention measure and used more by people in cluster 3. | High |
| Basic mental health care | 1% | Communication and coordination are necessary, named in the standard of care for patients with DM2, CVRM and COPD. | High |
| Specialist mental health care | 3% | Communication and coordination are necessary, named in the standard of care for patients with DM2, CVRM and COPD. However, it could be more difficult to connect between GP and mental healthcare specialist since these professions are further from each other. Also, a small number of patients uses care from the specialist mental health care. | Medium |
| Pharmacy | 98% | The standards of care point out that it is important that the pharmacist should be involved if multiple different medication is used by a patient. | High |
| (Medical) aids | 47% | The standard of care for COPD points out that it is important to monitor medical aids for breathing. Besides, the standards of care show the importance of aids that could stimulate the mobility of the patients. | High |
| District nursing | 13% | Especially important for elder patients. A high percentage of patients from clusters 5 and 6 use district nursing. Communication with district nurses was also mentioned in the standard of care for COPD. | High |
| Ophthalmology | 17% | Names in the standard of care for DM2. | High |
| Ear, nose, throat specialist (ENT) | 6% | Is not explicitly mentioned in the standards of care. It does not seem necessary to include this specialist in the bundle. However, a high percentage of patients uses care provided by this specialist. | Medium |
| Surgeon | 14% | Mentioned in the standard of care for DM2 (vascular surgeon). | High |
| Plastic surgeon | 3% | - | Low |
| Orthopaedics | 9% | Is not explicitly mentioned in the standards of care. It does not seem necessary to include this specialist in the bundle. However, a high percentage of patients uses care provided by this specialist. | Medium |
| Urology | 7% | Is not explicitly mentioned in the standards of care. It does not seem necessary to include this specialist in the bundle. However, a high percentage of patients uses care provided by this specialist. | Medium |
| Neurosurgeon | 1% | - | Low |
| Dermatology | 11% | Is not explicitly mentioned in the standards of care. It does not seem necessary to include this specialist in the bundle. However, a high percentage of patients uses care provided by this specialist. | Medium |
| Internal medicine | 13% | Mentioned in the standard of care for DM2 and CVR. | High |
| Gastro-enterology | 8% | Is not explicitly mentioned in the standards of care. It does not seem necessary to include this specialist in the bundle. However, a high percentage of patients uses care provided by this specialist. | Medium |
| Cardiology | 17% | Mentioned in the standard of care for DM2 and CVR. | High |
| Pulmonary disease | 9% | Mentioned in the standard of care for COPD. | High |
| Rheumatology | 4% | Is not explicitly mentioned in the standards of care. But 6% of the patients uses chronic medication for rheumatoid arthritis. Patients with rheumatoid arthritis could experience more difficulties with mobility. | Medium |
| Revalidation | 2% | Mentioned in the standard of care for COPD. Also relevant for people with and secondary cardiovascular disease. | High |
| Neurology | 10% | Mentioned in the standard of care for DM2. | High |
| Geriatrics | 3% | - | Low |
| Allergology | 0,1% | - | Low |
| Cardiopulmonary | 1% | Important for people with a combination of pulmonary problems and heart diseases. | High |
| Psychiatry | 0,4% | Communication with the psychiatrist is mentioned in the standard of care but only a very small number of patients uses care from this specialist. | Medium |
| Radiotherapy | 2% | - | Low |
| Radiology | 0,01% | - | Low |
| Anaesthesiology | 3% | - | Low |
| Clinical genetics | 0,2% | - | Low |
| Audiology | 0,3% | - | Low |
| Expensive medication | 3% | - | Low |
| Diagnostics | 90% | Diagnosis happens before someone is included in the bundle, which makes it difficult to include it in the bundle. But an adequate diagnosis is crucial for other (chronic) diseases and therefor important to be part of the bundle. | High |
| Medical specialist care at home | 2% | - | Low |
| Other products medical specialist care | 13% | Not possible to define which services are exactly covered by this sector. | Low |
| Primary care stay | 1% | - | Low |
| Geriatric rehabilitation care | 1% | - | Low |
| Other healthcare sectors | 9% | Not possible to define which services are exactly covered by this sector. | Low |
| Transport to the hospital | 9% | Impossible or very difficult to establish collaboration. | Low |

**Note: these arguments are based on the standards of care for DM2, COPD, and CVRM (NDF, 2015; NHG, 2020; LAN, 2013).*

## Appendix G. Risk of expanding the currently used SDMPs.

Table O provides insight into the ‘risk’ of extending the currently used SDMPs. The ‘risk’ is determined by how well the costs can be predicted for each care package. The unpredictability of the costs is where the risk lies. A profit for the PCC above €10 is highlighted in purple and a loss for each PCC above €10 is highlighted in blue to provide a quick overview of the (high) losses and profits.

Table O. Mean predicted profits or losses per primary care cooperative for the extension of the currently used SDMPs.

| Extend the current SDMPs with … | Observed Costs per patient* |  | Revenue or loss per patient per PCC | | |
| --- | --- | --- | --- | --- | --- |
|  |  |  | **Model 1:**  **Average (sd**)** | **Model 5:**  **Average (sd)** | **Model 7: Average (sd)** |
| No extension *(combination of the currently used SDMPs)* | €238 | *PCC 1*  *PCC 2*  *PCC 3* | €-36 (61)  €-4 (63)  €4 (60) | €-34 (69)  €-3 (69)  €4 (65) | €-33 (69)  €-3 (69)  €4 (65) |
| All general practitioners care (*without capitation payment, module nurse practitioner mental health care, and innovation and reward payment*) | €369 | *PCC 1*  *PCC 2*  *PCC 3* | €-38 (195)  €5 (186)  €-0.5 (192) | €-37 (185)  €4 (176)  €-0.3 (181) | €-36 (181)  €4 (176)  €-0.1 (179) |
| Physiotherapy | €435 | *PCC 1*  *PCC 2*  *PCC 3* | €-36 (448)  €21 (414)  €-9 (499) | €-35 (444)  €20 (405)  €-9 (491) | €-33 (443)  €19 (404)  €-9 (489) |
| Food therapy/ pedicure | €249 | *PCC 1*  *PCC 2*  *PCC 3* | €-36 (71)  €-4 (74)  €4 (72) | €-34 (78)  €-3 (79)  €4 (76) | €-33 (78)  €-4 (79)  €4 (76) |
| Ergotherapy | €246 | *PCC 1*  *PCC 2*  *PCC 3* | €-36 (81)  €-4 (84)  €4 (86) | €-34 (86)  €-3 (88)  €4 (89) | €-33 (87)  €-3 (88)  €4 (89) |
| Dietician | €242 | *PCC 1*  *PCC 2*  *PCC 3* | €-35 (66)  €-4 (69)  €4 (66) | €-33 (73)  €-3 (74)  €4 (70) | €-33 (73)  €-3 (74)  €4 (70) |
| Practice therapy | €245 | *PCC 1*  *PCC 2*  *PCC 3* | €-31 (68)  €-8 (132)  €6 (93) | €-29 (75)  €-8 (135)  €6 (96) | €-29 (75)  €-8 (135)  €6 (96) |
| Speech therapy | €241 | *PCC 1*  *PCC 2*  *PCC 3* | €-35 (74)  €-5 (128)  €5 (80) | €-33 (81)  €-4 (131)  €4 (84) | €-33 (82)  €-4 (131)  €4 (84) |
| Skin therapy | €239 | *PCC 1*  *PCC 2*  *PCC 3* | €-36 (68)  €-4 (64)  €4 (62) | €-34 (75)  3 (70)  €3 (66) | €-33 (75)  €-3 (70)  €4 (66) |
| Stop smoking modules | €240 | *PCC 1*  *PCC 2*  *PCC 3* | €-33 (76)  €-3 (73)  €3 (70) | €-33 (76)  €-3 (73)  €3 (70) | €-33 (77)  €-3 (73)  €3 (70) |
| Basic mental health care | €248 | *PCC 1*  *PCC 2*  *PCC 3* | €-36 (133)  €-3 (128)  €4 (128) | €-36 (133)  €-3 (128)  €4 (128) | €-36 (133)  €-3 (128)  €4 (128) |
| Specialist mental health care | €461 | *PCC 1*  *PCC 2*  *PCC 3* | €1 (2074)  €-1 (3359)  €1 (2950) | €-25 (2075)  €-1 (3358)  €2 (2949) | €-29 (2076)  €-1 (3358)  €2 (2949) |
| Pharmacy | €981 | *PCC 1*  *PCC 2*  *PCC 3* | €-55 (1996)  €30 (1292)  €-13 (2331) | €-63 (1952)  €27 (1237)  €-11 (2299) | €-61 (1942)  €25 (1229)  €-10 (2296) |
| (Medical) aids | €505 | *PCC 1*  *PCC 2*  *PCC 3* | €-18 (753)  €6 (871)  €-2 (830) | €-21 (726)  €5 (844)  €-2 (800) | €-22 (726)  €5 (844)  €-2 (800) |
| District nursing | €1.104 | *PCC 1*  *PCC 2*  *PCC 3* | €-24 (3668)  €-62 (3799)  €35 (3628) | €-59 (3286)  €-56 (3308)  €34 (3194) | €-59 (3286)  €-56 (3308)  €34 (3194) |
| Ophthalmology | €346 | *PCC 1*  *PCC 2*  *PCC 3* | €-18 (366)  €-13 (528)  €8 (458) | €-8 (372)  €-13 (533)  €7 (461) | €-7 (372)  €-13 (533)  €7 (461) |
| Ear, nose, throat specialist (ENT) | €272 | *PCC 1*  *PCC 2*  *PCC 3* | €-30 (205)  €-3 (323)  €3 (450) | €-28 (209)  €-2 (324)  €3 (451) | €-29 (209)  €-2 (324)  €3 (450) |
| Surgeon | €594 | *PCC 1*  *PCC 2*  *PCC 3* | €26 (1503)  €46 (1803)  €-27 (2243) | €3 (1489)  €46 (1788)  €-25 (2227) | €-17 (1472)  €48 (1760)  €-26 (2189) |
| Plastic surgeon | €269 | *PCC 1*  *PCC 2*  *PCC 3* | €-22 (183)  €-9 (326)  €6 (330) | €-20 (185)  €-9 (327)  €6 (331) | €-21 (185)  €-9 (327)  €6 (331) |
| Orthopaedics | €468 | *PCC 1*  *PCC 2*  *PCC 3* | €-61 (1632)  €2 (1468)  €-2 (1426) | €-66 (1626)  €2 (1461)  €3 (1420) | €-65 (1626)  €1 (1459)  €3 (1417) |
| Urology | €345 | *PCC 1*  *PCC 2*  *PCC 3* | €-59 (966)  €3 (794)  €1 (870) | €-60 (965)  €4 (794)  €1 (869) | €-64 (960)  €5 (791)  €1 (866) |
| Neurosurgeon | €268 | *PCC 1*  *PCC 2*  *PCC 3* | €-71 (1168)  €-19 (866)  €14 (580) | €-72 (1170)  €-19 (866)  €14 (580) | €-73 (1169)  €-19 (865)  €14 (580) |
| Dermatology | €282 | *PCC 1*  *PCC 2*  *PCC 3* | €-35 (221)  €6 (172)  €-1 (229) | €-31 (225)  €6 (175)  €-2 (230) | €-31 (225)  €6 (175)  €-2 (230) |
| Internal medicine | €515 | *PCC 1*  *PCC 2*  *PCC 3* | €18 (1345)  €21 (1820)  €-12 (2239) | €6 (1333)  €22 (1804)  €-12 (2224) | €-17 (1303)  €24 (1788)  €-12 (2195) |
| Gastro-enterology | €367 | *PCC 1*  *PCC 2*  *PCC 3* | €-36 (775)  €36 (510)  €-18 (991) | €-38 (774)  €36 (509)  €-17 (987) | €-43 (767)  €37 (507)  €-18 (980) |
| Cardiology | €552 | *PCC 1*  *PCC 2*  *PCC 3* | €-160 (2358)  €3 (1721)  €8 (1777) | €-167 (2335)  €-3 (1713)  €11 (1766) | €-180 (2317)  €-1 (1694)  €10 (1749) |
| Pulmonary disease | €423 | *PCC 1*  *PCC 2*  *PCC 3* | €-15 (1077)  €14 (1236)  €-7 (1317) | €-28 (1066)  €14 (1218)  €-6 (1302) | €-36 (1042)  €15 (1206)  €-6 (1287) |
| Rheumatology | €268 | *PCC 1*  *PCC 2*  *PCC 3* | €-43 (244)  €1 (232)  €2 (277) | €-41 (245)  €0.6 (233)  €2 (277) | €-40 (245)  €0.4 (233)  €2 (277) |
| Rehabilitation | €293 | *PCC 1*  *PCC 2*  *PCC 3* | €-56 (1331)  €-21 (1986)  €15 (865) | €-60 (1332)  €-22 (1986)  €15 (865) | €-67 (1330)  €-20 (1984)  €15 (864) |
| Neurology | €360 | *PCC 1*  *PCC 2*  *PCC 3* | €-74 (955)  €-6 (817)  €7 (771) | €-74 (949)  €-7 (812)  €8 (767) | €-82 (932)  €-6 (796)  €8 (754) |
| Geriatrics | €305 | *PCC 1*  *PCC 2*  *PCC 3* | €-48 (644)  €31 (267)  €-14 (806) | €-40 (642)  €31 (269)  €-15 (802) | €-43 (636)  €31 (270)  €-15 (793) |
| Allergology | €239 | *PCC 1*  *PCC 2*  *PCC 3* | €-36 (65)  €-4 (65)  €4 (64) | €-34 (72)  €-3 (70)  €4 (68) | €-34 (73)  €-3 (70)  €4 (68) |
| Cardiopulmonary | €301 | *PCC 1*  *PCC 2*  *PCC 3* | €-43 (1318)  €-3 (1503)  €4 (1192) | €-42 (1317)  €-3 (1502)  €4 (1191) | €-46 (1318)  €-2 (1500)  €3 (1190) |
| Psychiatry | €242 | *PCC 1*  *PCC 2*  *PCC 3* | €-33 (65)  €-1 (72)  €2 (108) | €-31 (72)  €-0,2 (77)  €2 (110) | €-31 (73)  €-0,2 (77)  €2 (110) |
| Radiotherapy | €292 | *PCC 1*  *PCC 2*  *PCC 3* | €-15 (433)  €-6 (706)  €4 (694) | €-16 (435)  €-5 (705)  €4 (694) | €-19 (434)  €-5 (702)  €4 (691) |
| Radiology | €238 | *PCC 1*  *PCC 2*  *PCC 3* | €-36 (62)  €-4 (68)  €4 (63) | €-33 (69)  €-3 (73)  €4 (67) | €-33 (69)  €-4 (73)  €4 (67) |
| Anaesthesiology | €277 | *PCC 1*  *PCC 2*  *PCC 3* | €-25 (236)  €9 (394)  €4 (591) | €-24 (239)  €9 (394)  €-4 (591) | €-25 (238)  €9 (394)  €-4 (590) |
| Clinical genetics | €240 | *PCC 1*  *PCC 2*  *PCC 3* | €-37 (89)  €-4 (81)  €4 (75) | €-34 (95)  €-4 (86)  €4 (78) | €-34 (95)  €-4 (86)  €4 (78) |
| Audiology | €239 | *PCC 1*  *PCC 2*  *PCC 3* | €-35 (64)  €-3 (66)  €4 (68) | €-33 (71)  €-3 (71)  €3 (72) | €-33 (71)  €-3 (72)  €3 (72) |
| Expensive medication | €444 | *PCC 1*  *PCC 2*  *PCC 3* | €34 (1524)  €18 (2350)  €-12 (3933) | €23 (1518)  €17 (2348)  €-10 (3931) | €-20 (1513)  €17 (2346)  €-10 (3929) |
| Diagnostics | €353 | *PCC 1*  *PCC 2*  *PCC 3* | €-1 (123)  €19 (169)  €-10 (167) | €4 (127)  €19 (170)  €-10 (168) | €4 (127)  €19 (170)  €-10 (167) |
| Medical specialist care at home | €272 | *PCC 1*  *PCC 2*  *PCC 3* | €-7 (89)  €-9 (485)  €5 (567) | €-9 (104)  €-8 (480)  €5 (565) | €-9 (108)  €-8 (481)  €5 (564) |
| Other products medical specialist care | €413 | *PCC 1*  *PCC 2*  *PCC 3* | €34 (811)  €25 (1869)  €-15 (2819) | €34 (813)  €25 (1865)  €-15 (2818) | €15 (815)  €27 (1852)  €-16 (2803) |
| Primary care stay | €301 | *PCC 1*  *PCC 2*  *PCC 3* | €-10 (330)  €14 (691)  €-7 (1103) | €-4 (336)  €14 (690)  €-8 (1098) | €-3 (333)  €14 (689)  €-7 (1098) |
| Geriatric rehabilitation care | €350 | *PCC 1*  *PCC 2*  *PCC 3* | €-102 (1651)  €2 (1489)  €7 (1445) | €-101 (1627)  €-1 (1481)  €6 (1438) | €-104 (1615)  €-1 (1470)  €6 (1428) |
| Other healthcare sectors | €268 | *PCC 1*  *PCC 2*  *PCC 3* | €-31 (248)  €3 (209)  €-0,03 (436) | €-32 (251)  €4 (210)  €-0,3 (436) | €-28 (251)  €3 (211)  €-0,3 (436) |
| Transport to the hospital | €324 | *PCC 1*  *PCC 2*  *PCC 3* | €-42 (350)  €-3 (362)  €4 (340) | €-42 (338)  €-3 (352)  €4 (332) | €-45 (328)  €-3 (341)  €4 (319) |

**Costs per patient are the real mean costs per patient of the currently used SDMPs combined with the named type of care, irrespective of utilization of care.*

***SD: standard deviation provides insight in the variation of revenue or loss on patient level within a primary care cooperative.*
